# Supplementary material for: An Innovative Deep Learning Approach for Ventilator-Associated Pneumonia (VAP) Prediction in Intensive Care Units—Pneumonia Risk Evaluation and Diagnostic Intelligence via Computational Technology (PREDICT)
Source: J Clin Med. 2025 May 13;14(10):3380. doi: 10.3390/jcm14103380 (PMC12112574; doi:10.3390/jcm14103380)
Supplement: Supplementary file 1 [file jcm-14-03380-s001.zip › Supplementary file A TRIPOD+AI Checklist.pdf]

Supplementary A. TRIPOD+AI Checklist

Table S1. TRIPOD+AI Checklist

**Section/Topic**	**Item**	**Development /Evaluation**	**Checklist Item**	**Reported on Page**
**TITLE**				
Title	1	D;E	Identify the study as developing or evaluating the performance of a multivariable prediction model, the target population, and the outcome to be predicted	Page 1
ABSTRACT				
Abstract	2	D;E	See TRIPOD+AI for Abstracts checklist	Page 1
INTRODUCTION				
Background	3a	D;E	Explain the healthcare context (including whether diagnostic or prognostic) and rationale for developing or evaluating the prediction model, including references to existing models	Page 1–2
	3b	D;E	Describe the target population and the intended purpose of the prediction model in the context of the care pathway, including its intended users (e.g., healthcare professionals, patients, public)	Page 2
	3c	D;E	Describe any known health inequalities between sociodemographic groups	NA
Objectives	4	D;E	Specify the study objectives, including whether the study describes the development or validation of a prediction model (or both)	Page 2
METHODS				
Data	5a	D;E	Describe the sources of data separately for the development and evaluation datasets (e.g., randomized trial, cohort, routine care or registry data), the rationale for using these data, and representativeness of the data	Page 2
	5b	D;E	Specify the dates of the collected participant data, including start and end of participant accrual; if applicable, end of follow-up	Page 2
Participants	6a	D;E	Specify key elements of the study setting (e.g., primary care, secondary care, general population) including the number and location of centres	Page 2
	6b	D;E	Describe the eligibility criteria for study participants	Page 3
	6c	D;E	Give details of any treatments received, and how they were handled during model development or evaluation, if relevant	Page 3
Data preparation	7	D;E	Describe any data pre-processing and quality checking, including whether this was similar across relevant sociodemographic groups	Page 4–5
Outcome	8a	D;E	Clearly define the outcome that is being predicted and the time horizon, including how and when it was assessed, the rationale for choosing this outcome, and whether the method of outcome assessment is consistent across sociodemographic groups	Page 3
	8b	D;E	If outcome assessment requires subjective interpretation, describe the qualifications and demographic characteristics of the outcome assessors	NA

	8c	D;E	Report any actions to ensure blind assessment of the outcome to be predicted	
Predictors	9a	D	Describe the choice of initial predictors (e.g., the literature, previous models, all available predictors) and any pre-selection of predictors before model building	Page 4
	9b	D;E	Clearly define all predictors, including how and when they were measured (and any actions to ensure blind assessment of predictors for the outcome and other predictors)	Page 4
	9c	D;E	If predictor measurement requires subjective interpretation, describe the qualifications and demographic characteristics of the predictor assessors	NA
Sample size	10	D;E	Explain how the study size was arrived at (separately for development and evaluation), and justify that the study size was sufficient to answer the research question. Include details of any sample size calculation	NA
Missing data	11	D;E	Describe how missing data were handled. Provide reasons for omitting any data	Page 4
Analytical methods	12a	D	Describe how the data were used (e.g., for development and evaluation of model performance) in the analysis, including whether the data were partitioned, considering any sample size requirements	Page 4–5
	12b	D	Depending on the type of model, describe how predictors were handled in the analyses (functional form, rescaling, transformation, or any standardization).	Page 4–5
	12c	D	Specify the type of model, rationale, all model-building steps, including any hyperparameter tuning, and method for internal validation	Page 5 + supplementary file B
	12d	D;E	Describe if and how any heterogeneity in estimates of model parameter values and model performance was handled and quantified across clusters (e.g., hospitals, countries). See TRIPOD-Cluster for additional considerations	
	12e	D;E	Specify all measures and plots used (and their rationale) to evaluate model performance (e.g., discrimination, calibration, clinical utility) and, if relevant, to compare multiple models	Page 5–6 + supplementary file B
	12f	E	Describe any model updating (e.g., recalibration) arising from the model evaluation, either overall or for particular sociodemographic groups or settings	
	12g	E	For model evaluation, describe how the model predictions were calculated (e.g., formula, code, object, application programming interface)	Page 6
Class imbalance	13	D;E	If class imbalance methods were used, state why and how this was done, and any subsequent methods to recalibrate the model or the model predictions	Page 5
Fairness	14	D;E	Describe any approaches that were used to address model fairness and their rationale	
Model output	15	D	Specify the output of the prediction model (e.g., probabilities, classification). Provide details and rationale for any classification and how the thresholds were identified	Page 5
Training versus evaluation	16	D;E	Identify any differences between the development and evaluation data in healthcare setting, eligibility criteria, outcome, and predictors	
Ethical approval	17	D;E	Name the institutional research board or ethics committee that approved the study and describe the participant-	Page 12–13

			informed consent or the ethics committee waiver of informed consent	
OPEN SCIENCE				
Funding	18a	D;E	Give the source of funding and the role of the funders for the present study	Page 12–13
Conflicts of interest	18b	D;E	Declare any conflicts of interest and financial disclosures for all authors	Page 13
Protocol	18c	D;E	Indicate where the study protocol can be accessed or state that a protocol was not prepared	NA
Registration	18d	D;E	Provide registration information for the study, including register name and registration number, or state that the study was not registered	Page 13
Data sharing	18e	D;E	Provide details of the availability of the study data	Page 12–13
Code sharing	18f	D;E	Provide details of the availability of the analytical code	Page 13
PATIENT & PUBLIC INVOLVEMENT				
Patient & Public Involvement	19	D;E	Provide details of any patient and public involvement during the design, conduct, reporting, interpretation, or dissemination of the study or state no involvement.	
RESULTS				
Participants	20a	D;E	Describe the flow of participants through the study, including the number of participants with and without the outcome and, if applicable, a summary of the follow-up time. A diagram may be helpful.	Page 6
	20b	D;E	Report the characteristics overall and, where applicable, for each data source or setting, including the key dates, key predictors (including demographics), treatments received, sample size, number of outcome events, follow-up time, and amount of missing data. A table may be helpful. Report any differences across key demographic groups.	Page 6–7
	20c	E	For model evaluation, show a comparison with the development data of the distribution of important predictors (demographics, predictors, and outcome).	Page 8–9
Model development	21	D;E	Specify the number of participants and outcome events in each analysis (e.g., for model development, hyperparameter tuning, model evaluation)	Supplementary file B
Model specification	22	D	Provide details of the full prediction model (e.g., formula, code, object, application programming interface) to allow predictions in new individuals and to enable third-party evaluation and implementation, including any restrictions to access or re-use (e.g., freely available, proprietary)	Supplementary file B
Model performance	23a	D;E	Report model performance estimates with confidence intervals, including for any key subgroups (e.g., sociodemographic). Consider plots to aid presentation.	Page 8 + supplementary file D
	23b	D;E	If examined, report results of any heterogeneity in model performance across clusters. See TRIPOD-Cluster for additional details.	
Model updating	24	E	Report the results from any model updating, including the updated model and subsequent performance	
DISCUSSION				
Interpretation	25	D;E	Give an overall interpretation of the main results, including issues of fairness in the context of the objectives and previous studies	Page 11–12

Limitations	26	D;E	Discuss any limitations of the study (such as a non-representative sample, sample size, overfitting, missing data) and their effects on any biases, statistical uncertainty, and generalizability	Page 12
Usability of the model in the context of current care	27a	D	Describe how poor quality or unavailable input data (e.g., predictor values) should be assessed and handled when implementing the prediction model	Page 12
	27b	D	Specify whether users will be required to interact in the handling of the input data or use of the model, and what level of expertise is required of users	
	27c	D;E	Discuss any next steps for future research, with a specific view to applicability and generalizability of the model	Page 12
