# Supplementary material for: An Innovative Deep Learning Approach for Ventilator-Associated Pneumonia (VAP) Prediction in Intensive Care Units—Pneumonia Risk Evaluation and Diagnostic Intelligence via Computational Technology (PREDICT)
Source: J Clin Med. 2025 May 13;14(10):3380. doi: 10.3390/jcm14103380 (PMC12112574; doi:10.3390/jcm14103380)
Supplement: Supplementary file 1 [file jcm-14-03380-s001.zip › Supplementary file B Model Development.pdf]

Supplementary B. Model Development

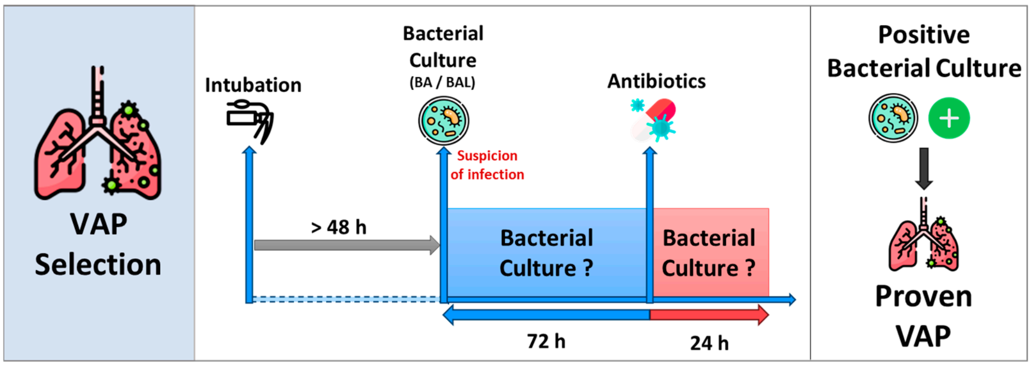

Figure S1. VAP annotation algorithm.

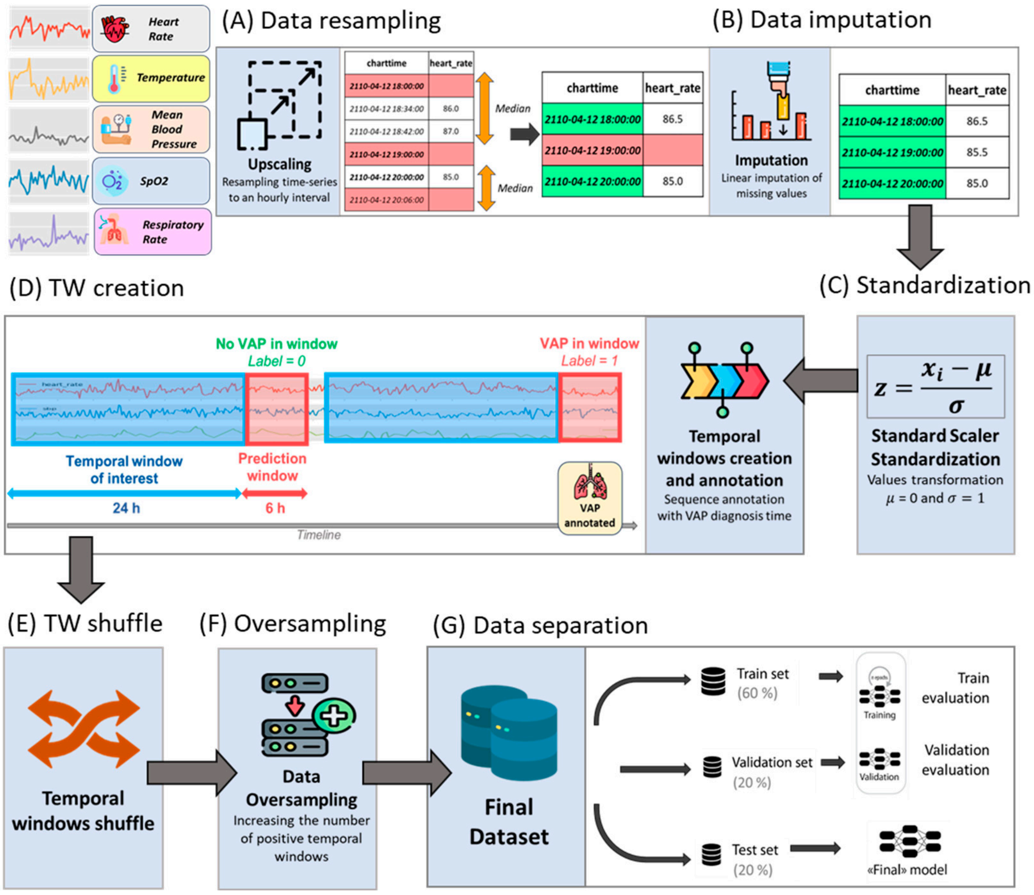

Figure S2. Data preprocessing pipeline.

Table S2. Data separation details.

Temporal Windows with Oversampling (*n*)	Train Set	Validation Set	Test Set
VAP 6 h prediction	207,960	69,320	69,320
VAP 12 h prediction	202,954	67,652	67,652
VAP 24 h prediction	196,708	65,570	65,570

Supplementary B.1. Data Oversampling

VAP temporal windows (TWs) represented less than 1% of all TWs in the dataset. To improve model performance in this case of unbalanced learning, we have chosen, as recommended, to implement an oversampling strategy [18]. We used the synthetic minority oversampling technique (SMOTE) [17], a method used to balance class distribution by generating synthetic samples of the minority class. The core idea was to create synthetic examples along the line segments joining a minority class instance and its nearest neighbors. An empirically derived oversampling rate of 3% of the total TW for the minority class was selected for VAP prediction windows at 6 and 12 h. An oversampling rate of 5% was used for VAP prediction windows at 24 h as TWs already represent 3% of dataset for 24 h predictions (supplementary Table S2). This process involved 3 key stages: (1) Each TW was flattened for conversion to a dataset shape the technique can handle (vectors with shape [x, 24 × 5 = 120] with x = number of TW). (2) SMOTE technique was then applied to the flattened dataset. (3) The sequences were restored to their original configuration (array with shape [x, 24, 5]—Figure). We assessed the quality of the oversampling strategy by studying a graphical representation of a sample of 100 augmented TWs to ensure that the chronological logic of the time-series was respected.

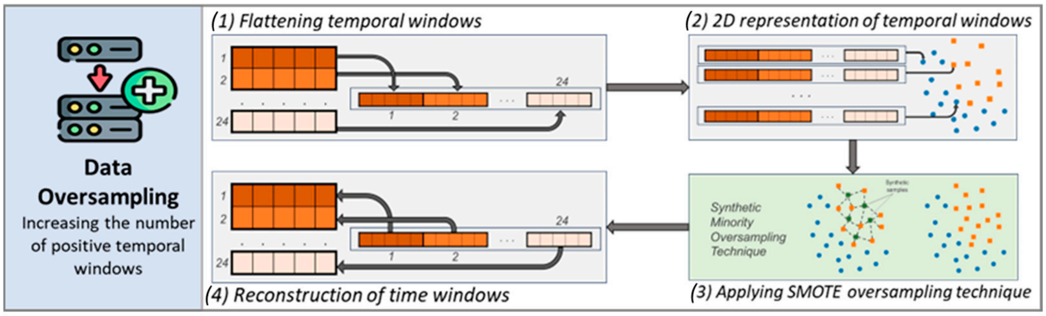

Figure S3. Data oversampling technique.

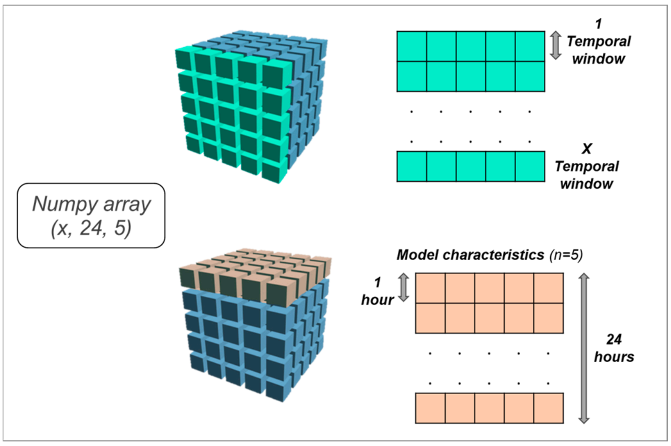

Figure S4. 3D data representation for algorithm training.

Table S3. Temporal windows before and after oversampling.

Temporal Windows (*n*)	Initial VAP Temporal Windows	% Before Oversampling	Oversampled PAVM Temporal Windows	% After Oversampling
**VAP 6 h prediction**	2260	0.6	10,095	3
**VAP 12 h prediction**	4965	1.5	9852	3
**VAP 24 h prediction**	10,347	3.2	14,630	5

Supplementary B.2. Model Development

For algorithm regularization, we used two techniques: dropout and batch-normalization. Batch normalization layers were inserted between each LSTM layer ($n = 3$) in order to stabilize and accelerates the learning process by normalizing the outputs of the LSTM layers [34,35]. Additionally, it acted as a regularizer, introducing slight noise during training which mitigates overfitting and improves the model's generalization capability. To enhance the training, we used a custom optimizer based on the Adam algorithm. This optimizer incorporated adaptive learning rate schedules and weight decay to improve the convergence speed and model stability. Weight decay acted as a regularizer, preventing overfitting by penalizing large weights [36]. The optimizer employed momentum, which leveraged past gradients to smooth the optimization trajectory and accelerate convergence [37]. For each time horizon, the number of training epochs was optimized using early stopping based on validation loss. This approach minimized overfitting and allowed dynamic adaptation to the temporal prediction difficulty. Hyperparameter optimization (HPO) was manually realized with the study of training curves after each train. The hyperparameters that could be varied were the dropout rate, the number of layers, and the number of neurons in each layer. The evaluation of the model metrics during the HPO process was conducted using the validation set. After each training session, a precision–recall curve was plotted. The classification threshold selected for evaluating the performance of the algorithm was the crossover point offering both the best possible precision and recall.

Concerning ML algorithms, for each prediction window (6, 12, and 24 h), the HPO procedure required the training of 135 XGBoost models, 135 LightGBM models, 45 random forest models, and 15 logistic regression models. HPO was carried out to optimize the AUPRC, which is highly effective when working with unbalanced data. The hyperparameters included in the HPO procedure are listed in the following supplementary data.

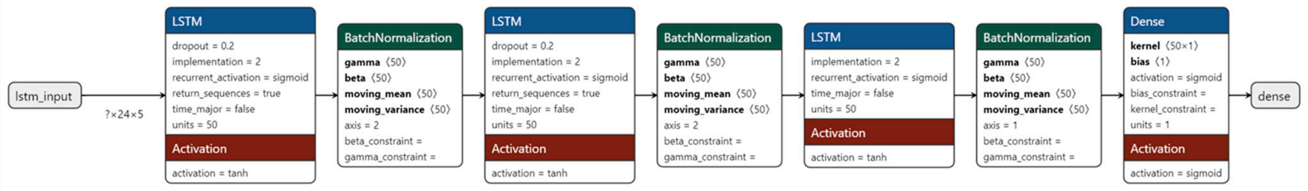

Figure S5. PREDICT deep learning final model architecture.

Table S4. Hyperparameters used for HPO procedure in concurrent ML models' development.

Model	Hyperparameter	Values
**XGBoost**	n_estimators	50, 100, 200
	max_depth	3, 6, 9
	learning_rate	0.01, 0.1, 0.2
**LightGBM**	n_estimators	50, 100, 200
	max_depth	−1, 10, 20
	learning_rate	0.01, 0.1, 0.2
**RandomForest**	n_estimators	50, 100, 200
	max_depth	10, 20, 30
**LogisticRegression**	C	0.1, 1, 10
	penalty	l2
