# Supplementary material for: An Innovative Deep Learning Approach for Ventilator-Associated Pneumonia (VAP) Prediction in Intensive Care Units—Pneumonia Risk Evaluation and Diagnostic Intelligence via Computational Technology (PREDICT)
Source: J Clin Med. 2025 May 13;14(10):3380. doi: 10.3390/jcm14103380 (PMC12112574; doi:10.3390/jcm14103380)
Supplement: Supplementary file 1 [file jcm-14-03380-s001.zip › Supplementary file C Metrics Definition.pdf]

Journal of Clinical Medicine

Supplementary C. Metrics Definition

Supplementary C.1. Positive Predictive Value (PPV) or Precision

Precision is the ratio of correctly predicted positive observations to the total predicted positives. It is a measure of the accuracy of the positive predictions.

$$Precision\ or\ PPV = \frac{TP}{TP + FP}$$

Supplementary C.2. Sensibility or Recall

Sensibility is the ratio of correctly predicted positive observations to all the observations in the actual class.

$$Sensibility = \frac{TP}{TP + FN}$$

Supplementary C.3. Negative Predictive Value (NPV)

NPV is the ratio of correctly predicted negative observations to the total predicted negatives. It measures how well the model predicts negative cases.

$$NPV = \frac{TN}{TN + FN}$$

Supplementary C.4. Specificity (True Negative Rate)

Specificity is the ratio of correctly predicted negative observations to all the observations in the actual negative class. It measures the proportion of actual negatives that are correctly identified.

$$Specificity = \frac{TN}{TN + FP}$$

Supplementary C.5. Youden Index

The Youden Index is a statistical measure used to evaluate the effectiveness of a diagnostic test. It is particularly useful when assessing the trade-off between sensitivity and specificity. The index provides a single summary statistic that captures the test's overall ability to distinguish between positive and negative cases.

$$Youden\ Index = Sensitivity + Specificity - 1$$

Supplementary C.6. Area Under the Receiver Operating Characteristic Curve (AUROC)

The AUROC measures the ability of a classifier to distinguish between positive and negative classes across all possible classification thresholds. It is computed as the area under the receiver operating characteristic (ROC) curve, which plots the true positive rate (TPR) (sensitivity) against the false positive rate (FPR) for different thresholds. The AUROC ranges from 0.5 (no discriminatory ability, equivalent to random guessing) to 1.0 (perfect classifier). Higher AUROC values indicate better performance in distinguishing between classes.

$$AUROC = \int_0^1 TPR\left(FPR^{-1}(x)\right)dx$$

where:

$$TPR = \frac{True\ Positives}{True\ Positives + False\ Negatives}$$

$$FPR = \frac{False\ Positives}{False\ Positives + True\ Negatives}$$

Supplementary C.7. Area Under the Precision-Recall Curve (AUPRC)

The AUPRC evaluates the classifier's performance by measuring the area under the precision–recall (PR) curve, which plots the precision against the recall at various thresholds. It is particularly suitable for imbalanced datasets, where the positive class is rare. The AUPRC ranges from the prevalence of the positive class (baseline performance for random predictions) to 1.0 (perfect classifier). It emphasizes the balance between precision and recall, making it more informative than AUROC in cases of class imbalance.

$$AUPRC = \int_0^1 Precision\big(Recall^{-1}(x)\big)dx$$

where:

$$Precision = \frac{True\ Positives}{True\ Positives\ +\ False\ Positives}$$

$$Recall\ (or\ TPR)\ = \frac{True\ Positives}{True\ Positives\ +\ False\ Negatives}$$
