# Supplementary material for: An Innovative Deep Learning Approach for Ventilator-Associated Pneumonia (VAP) Prediction in Intensive Care Units—Pneumonia Risk Evaluation and Diagnostic Intelligence via Computational Technology (PREDICT)
Source: J Clin Med. 2025 May 13;14(10):3380. doi: 10.3390/jcm14103380 (PMC12112574; doi:10.3390/jcm14103380)
Supplement: Supplementary file 1 [file jcm-14-03380-s001.zip › Supplementary file D Training Curves and Confusion Matrix.pdf]

Supplementary D. Training Curves and Confusion Matrix

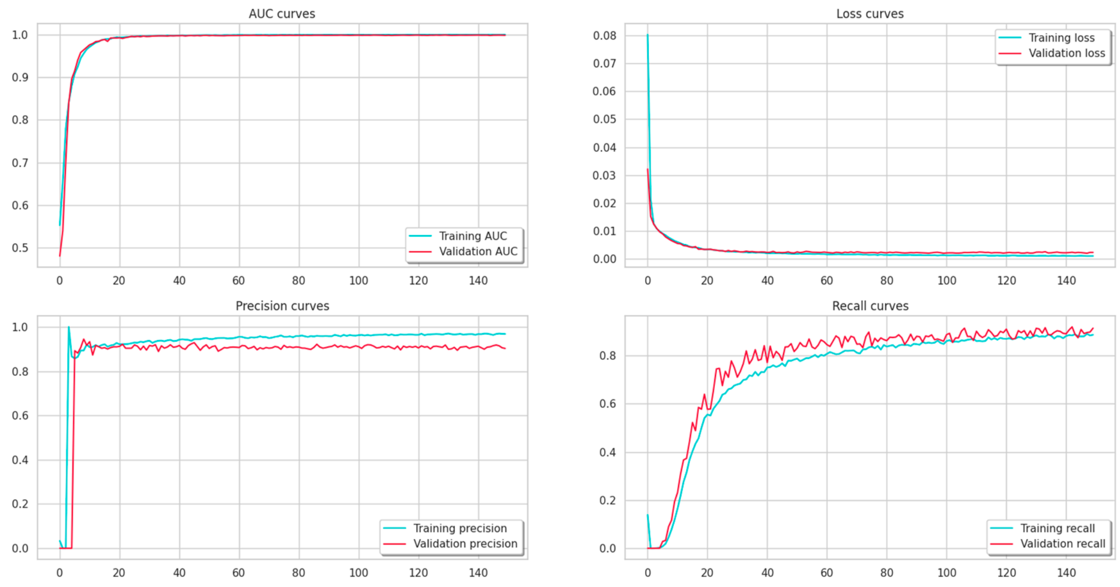

Figure S6. Training curves for PREDICT 6 h.

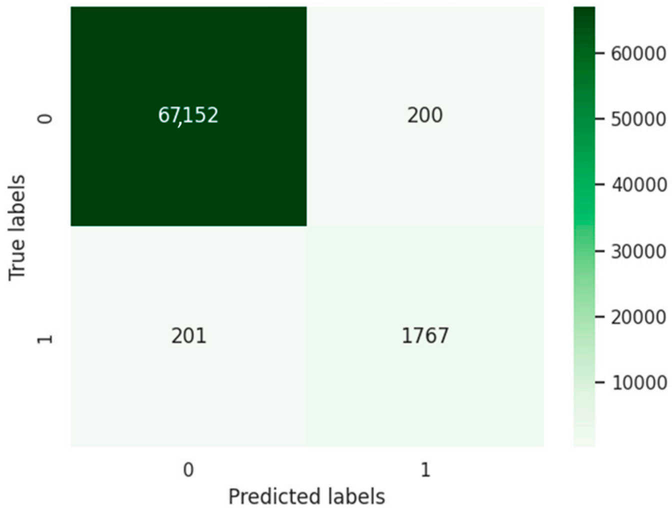

Figure S7. Confusion matrix for PREDICT 6 h.

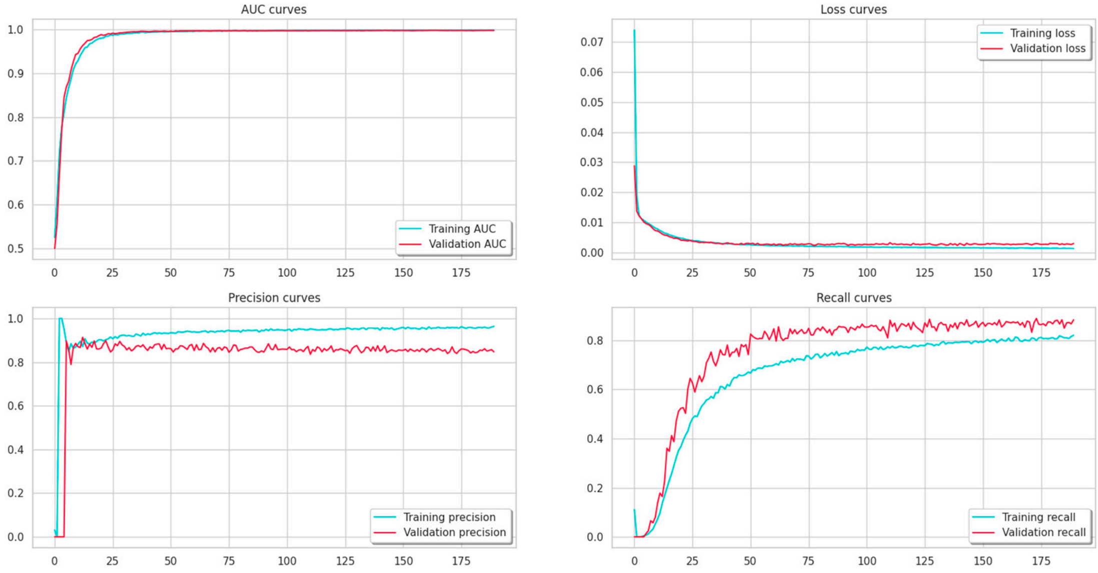

Figure S8. Training curves for PREDICT 12 h.

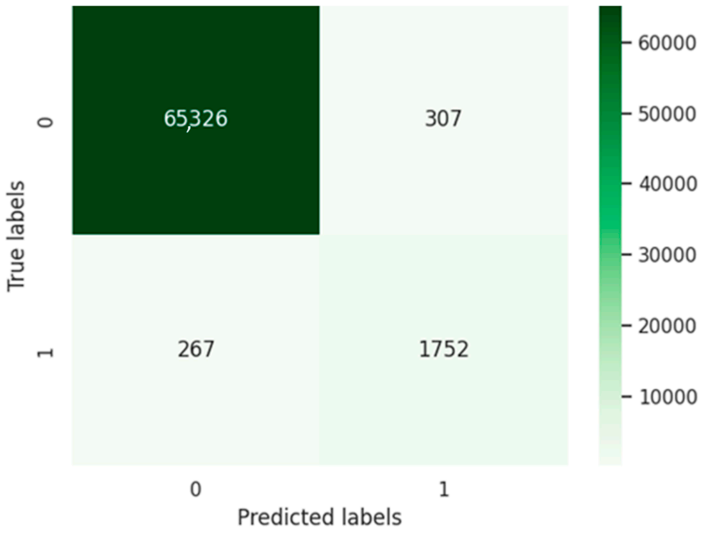

Figure S9. Confusion matrix for PREDICT 12 h.

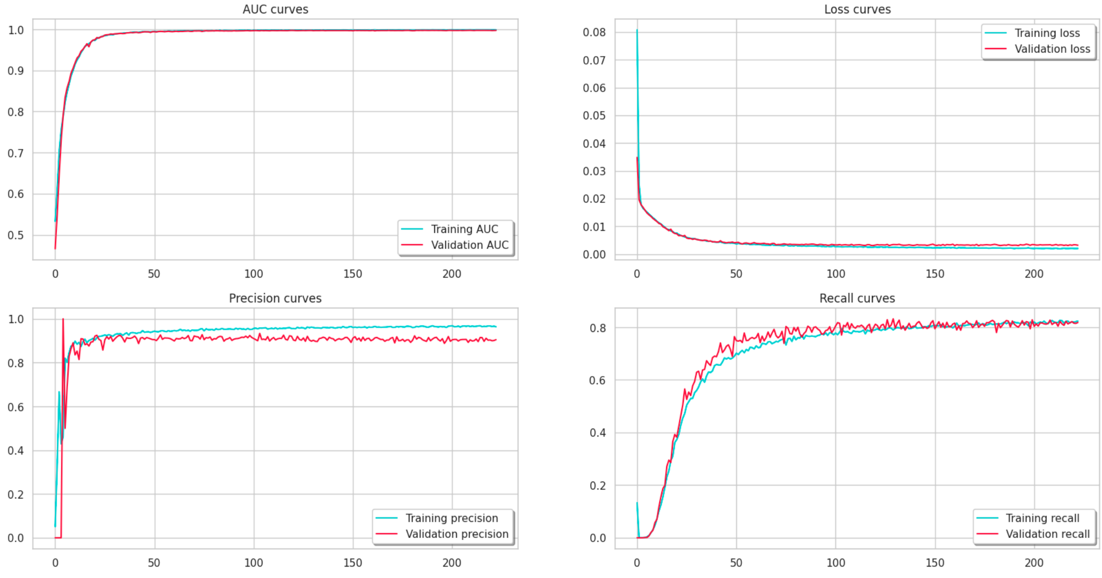

Figure S10. Training curves for PREDICT 24 h.

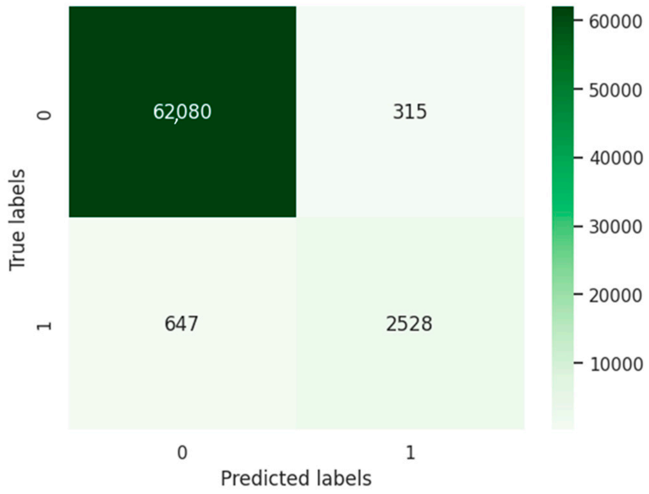

Figure S11. Confusion matrix for PREDICT 24 h.

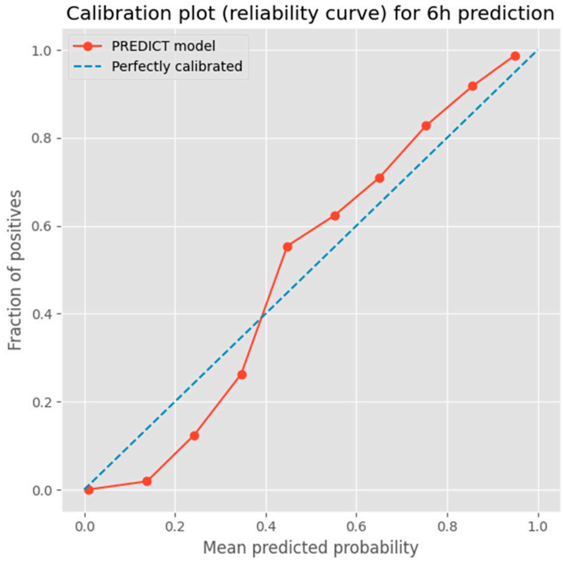

Figure S12. Calibration plot for PREDICT algorithm for 6 h VAP prediction.

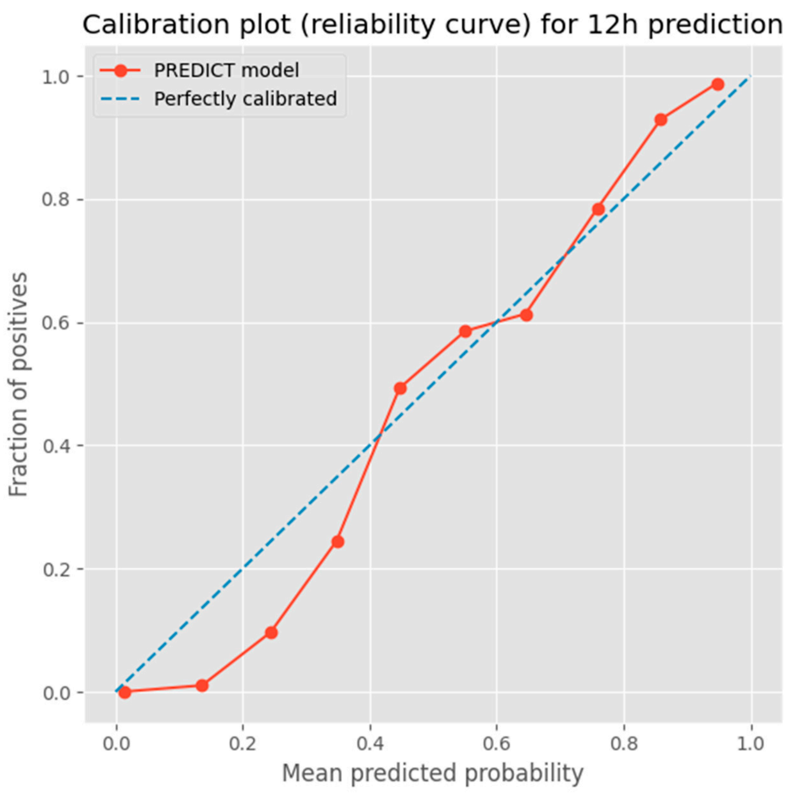

Figure S13. Calibration plot for PREDICT algorithm for 12 h VAP prediction.

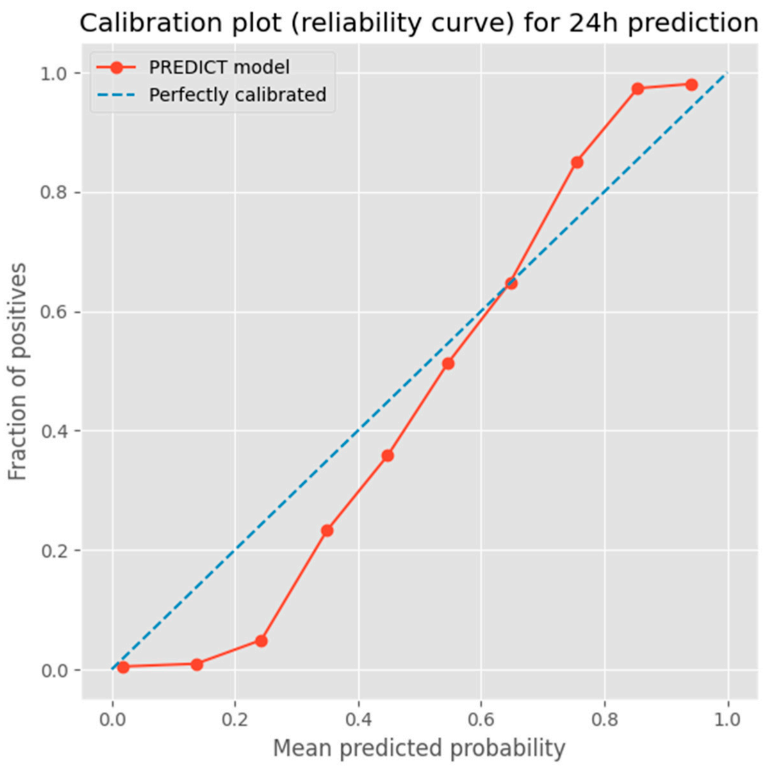

Figure S14. Calibration plot for PREDICT algorithm for 24 h VAP prediction.
