# Supplementary material for: An Innovative Deep Learning Approach for Ventilator-Associated Pneumonia (VAP) Prediction in Intensive Care Units—Pneumonia Risk Evaluation and Diagnostic Intelligence via Computational Technology (PREDICT)
Source: J Clin Med. 2025 May 13;14(10):3380. doi: 10.3390/jcm14103380 (PMC12112574; doi:10.3390/jcm14103380)
Supplement: Supplementary file 1 [file jcm-14-03380-s001.zip › Supplementary file E Metrics Comparison.pdf]

Journal of Clinical Medicine

MDPI

Supplementary E. Metrics Comparison

Table S5. Complete model metrics with 95% confidence intervals.

Algorithm	VAP Prediction	Best Threshold	AUROC	AUPRC	Sensibility	Specificity	PPV	NPV	Youden-Index
**XGBoost**	**6 h**	0.03	0.992 (0.99–0.994)	0.941 (0.933–0.949)	0.887 (0.875–0.9)	0.997 (0.996–0.997)	0.887 (0.874–0.901)	0.997 (0.996–0.997)	0.884 (0.872–0.897)
	**12 h**	0.04	0.956 (0.95–0.961)	0.744 (0.726–0.757)	0.713 (0.692–0.73)	0.991 (0.99–0.992)	0.711 (0.691–0.727)	0.991 (0.99–0.992)	0.704 (0.683–0.721)
	**24 h**	0.07	0.915 (0.909–0.919)	0.614 (0.597–0.632)	0.596 (0.578–0.611)	0.979 (0.978–0.981)	0.596 (0.581–0.612)	0.979 (0.978–0.98)	0.575 (0.558–0.591)
**LightGBM**	**6 h**	0.09	0.964 (0.959–0.969)	0.857 (0.845–0.873)	0.807 (0.792–0.824)	0.994 (0.994–0.995)	0.808 (0.789–0.827)	0.994 (0.994–0.995)	0.801 (0.786–0.818)
	**12 h**	0.07	0.891 (0.884–0.899)	0.597 (0.576–0.615)	0.556 (0.534–0.577)	0.986 (0.985–0.987)	0.556 (0.538–0.576)	0.986 (0.985–0.987)	0.542 (0.52–0.563)
	**24 h**	0.09	0.822 (0.813–0.831)	0.492 (0.474–0.509)	0.452 (0.433–0.471)	0.972 (0.971–0.973)	0.452 (0.436–0.471)	0.972 (0.971–0.973)	0.424 (0.406–0.443)
**Random Forest**	**6 h**	0.12	0.993 (0.99–0.995)	0.917 (0.904–0.93)	0.905 (0.894–0.917)	0.997 (0.997–0.998)	0.906 (0.893–0.918)	0.997 (0.997–0.998)	0.902 (0.891–0.915)
	**12 h**	0.12	0.957 (0.951–0.963)	0.68 (0.654–0.701)	0.761 (0.741–0.776)	0.993 (0.992–0.993)	0.759 (0.738–0.776)	0.993 (0.992–0.993)	0.754 (0.733–0.769)
	**24 h**	0.13	0.909 (0.902–0.914)	0.54 (0.519–0.566)	0.627 (0.611–0.646)	0.981 (0.98–0.982)	0.627 (0.61–0.643)	0.981 (0.98–0.982)	0.608 (0.592–0.626)
**Logistic Regression**	**6 h**	0.05	0.599 (0.587–0.611)	0.037 (0.035–0.04)	0.036 (0.029–0.044)	0.972 (0.971–0.973)	0.036 (0.029–0.044)	0.972 (0.971–0.973)	0.008 (0.001–0.016)
	**12 h**	0.05	0.586 (0.573–0.601)	0.04 (0.038–0.043)	0.051 (0.044–0.061)	0.971 (0.97–0.972)	0.051 (0.044–0.061)	0.971 (0.97–0.972)	0.022 (0.014–0.031)
	**24 h**	0.07	0.557 (0.548–0.565)	0.057 (0.054–0.059)	0.061 (0.054–0.07)	0.952 (0.951–0.954)	0.061 (0.053–0.07)	0.952 (0.951–0.954)	0.013 (0.006–0.022)

Table S6. Balanced accuracy and Matthews correlation coefficient for PREDICT algorithm.

	VAP Prediction	Balanced Accuracy	Matthews Correlation Coefficient
**PREDICT Model**	6 h	0.947 [0.941, 0.955]	0.895 [0.885, 0.909]
	12 h	0.923 [0.914, 0.928]	0.849 [0.838, 0.859]
	24 h	0.886 [0.881, 0.891]	0.83 [0.822, 0.836]
